# Experiences of Persons With Disabilities Regarding Home Adaptation and Assistive Products in a Rural Community of Northern Thailand: Qualitative Study

**DOI:** 10.2196/95026

**Published:** 2026-06-29

**Authors:** Anuchart Kaunnil, Hataichanok Apikomonkon, Jiranan Griffiths, Peeraya Munkhetvit

**Affiliations:** 1 Department of Occupational Therapy, Faculty of Associated Medical Sciences, Chiang Mai University, 110 Intawaroros Rd, Sripoom Subdistrict, Mueang Chiang Mai District, Chiang Mai, 50180, Thailand, 66 53949252, 66 53936042

**Keywords:** persons with disabilities, home adaptations, assistive products, rural community, occupational therapy

## Abstract

**Background:**

Chiang Mai Province, located in northern Thailand, is a well-known tourist destination. Its rural areas are characterized by mountainous and valley landscapes, where residents primarily engage in agricultural activities and maintain traditional ways of life rooted in Northern Thai culture. However, persons with disabilities in rural areas of Chiang Mai face significant challenges in enhancing their quality of life and well-being. Home adaptations and assistive products are essential for these individuals, as they facilitate daily activities and improve overall living conditions. To address the needs of persons with disabilities in these communities effectively, it is crucial to gather insights and perspectives that reflect their everyday experiences.

**Objective:**

This study aimed to explore the experiences of persons with disabilities, focusing on the needs and obstacles they encounter in accessing home adaptation and using assistive products.

**Methods:**

A qualitative approach was used for this study. Individual interviews were conducted with 11 persons with disabilities residing in rural communities of the Doilor Subdistrict in Chiang Mai, Thailand. The data were analyzed using thematic analysis.

**Results:**

Five themes emerged: (1) financial and personal burdens of home adaptations, (2) living with safety risks in the home environment, (3) the need for assistive products aligned with individual health conditions and contexts, (4) challenges in accessing mobility support and essential services, and (5) emotional impact and sociocultural support.

**Conclusions:**

The findings indicate that persons with disabilities in rural northern Thailand encounter multifaceted barriers to enhancing their mobility and adapting to their home environments. More effort is needed to enhance home modification support, integrate assistive products, and provide transportation accessibility that promotes community inclusion for persons with disabilities. This study highlights the influence of organizational priorities on community practice and the need for person-centered practice to ensure that modifications lead to meaningful outcomes valued by persons with disabilities in the future.

## Introduction

### Background

In 2024, Thailand had approximately 2.18 million persons with disabilities, with 51.96% (1,136,174/2,186,769) being male and 48.04% (1,050,595/2,186,769) being female. The most common types of disabilities are physical or mobility disabilities (1,106,553/2,186,769, 50.60%), visual impairment (177,999/2,186,765, 8.43%), and intellectual disabilities (150,688/2,186,765, 6.86%). Most of them have graduated primary school (1,400,625/2,186,765, 64.05%) and live in rural areas, with the highest proportions residing in the Northeast (879,081/2,186,765, 40.20%) and North (508,423/2,186,765, 23.25%) regions [1]. While the employment of persons with disabilities is low, the ratio of employees with disabilities to no disabilities is approximately 1:100 [2]. The National Statistical Office (NSO) and the United Nations Children’s Fund reported that in 2022, the total population of persons with disabilities was 4,192,291 or 6% of the entire Thai population. This represents a significant increase from 5.5% in 2017 [3]. Although the Empowerment of Persons With Disabilities Act and the National Development Plan for the Empowerment of Persons With Disabilities (Fifth Edition) aim to support all persons with disabilities, the national committee established by this legislation provides only state aid to Thai citizens with identification and disability cards [4].

According to the 2022 report by the NSO Thailand, 99.1% (2,134,137/2,153,519) of persons with disabilities can access universal health care services under national welfare schemes. However, only 42.6% (917,399/2,153,519) are registered for disability certification, indicating that 57.4% (1,236,119/2,153,519) do not receive formal financial support and resources [5]. Additionally, 4.1% (88,294/2,153,519) are unable to access required medical treatment, 9.2% (198,123/2,153,519) require rehabilitation services, and 16.6% (357,484/2,153,519) lack essential prosthetic or assistive devices, equivalent to approximately 696,000 individuals. The most commonly unmet needs included canes, wheelchairs, forearm crutches, hearing aids, and eyeglasses [5]. Rural-urban disparities in access to services for persons with disabilities in Thailand remain insufficiently quantified in the studies. Nonetheless, reports from the NSO Thailand found that persons with disabilities in rural areas experience significant support gaps, including long travel distances and limited availability of health care professionals and services. Given Thailand’s geographic landscape, rural areas are likely to have fewer rehabilitation facilities and assistive product providers per capita [5]. Consequently, individuals experiencing significant hardship are often underrepresented in national surveys. For example, ethnic hill tribe populations living in remote forest and mountainous areas, as well as individuals born in rural communities without Thai citizenship, are frequently overlooked and may be unable to obtain disability cards, thereby limiting their access to services and support [6].

In Thailand, it is a predominantly Buddhist country, with approximately 93% of the population adhering to this faith [7]. Thai Buddhist culture is deeply rooted in the practice of merit making, which is based on the fundamental beliefs of karma (human actions) and destiny. The concept of karma, or the belief that actions in a previous time or past life influence one’s current circumstances and future, is a central tenet of Thai Buddhism [8]. Health and illness in Buddhism reflect interdependence and cause-and-effect relationships in life. Moreover, the Thai Buddhist belief that “doing good will get good, doing bad will get bad” has influenced the cultural understanding of disability [9]. They believe that this legacy is inherited from devil karma and the sins of past lives. This belief leads to the view that disability is a personally earned tragedy, embodying faults accumulated in past or present lives. This cultural perspective often results in sympathy or mercy toward disabled people, while societal responsibility for ensuring their rights is overlooked [10]. Hence, the stigma surrounding disability in Thai society was found to hinder access to opportunities for persons with disabilities, particularly in rural areas.

In rural areas of Chiang Mai, Northern Thailand, vernacular stilt housing constitutes a distinctive regional architectural style that is deeply embedded in local sociocultural practices and ways of life. These dwellings are typically characterized by elevated structures, designed to reduce exposure to ground-level cold and moisture, reflecting adaptive responses to the local environmental context. These dwellings commonly incorporate architectural features such as external stair access, steeply pitched roofs, and detached sanitation facilities located at a distance from the main house for practical and hygienic purposes [11]. Moreover, road infrastructure and public transportation in rural Northern Thailand demonstrate marked spatial disparities between lowland and upland areas. In upland areas, road infrastructures remain uneven and inadequate. Many villages are on narrow concrete or unpaved roads that become impassable during the rainy season, limiting access to health care, education, and economic opportunities. In contrast, lowland areas have found well-developed, all-weather roads with reliable links to urban centers. Public transportation in upland areas is not available, with residents depending primarily on private vehicles, highlighting persistent inequities in transportation access [12].

Home adaptation and the use of assistive products can help persons with disabilities overcome challenges in daily activities, promoting greater independence and a better quality of life [13]. However, persons with disabilities encounter limited access to health care support services, such as home adaptations and assistive devices. There is an urgent need to enhance national policies to improve these services [14]. Additionally, the lack of specific guidelines for housing adaptations hinders the implementation of policies aimed at improving the quality of life for persons with disabilities [15]. Furthermore, persons with disabilities in rural areas often face distinct challenges that differ from those experienced by persons with disabilities in urban settings. These unique challenges are influenced by various cultural and contextual factors, which can hinder the full implementation of policies or limit the advocacy for the rights of persons with disabilities [16]. Living with a disability in rural areas can be a challenging experience because various factors can significantly impact the quality of life of those affected. The geographic environment and the lack of infrastructure, transportation, specialized health care, and rehabilitation services in rural areas can create significant barriers to independence, social integration, and overall well-being for persons with disabilities [17].

### Literature Review

Home adaptations refer to modifications made to the built environment to enhance safety, accessibility, and usability for older adults and persons with disabilities [18]. They are widely recognized as effective interventions that support independent living and reduce reliance on formal or informal caregiving [19,20]. In aging societies, the home environment plays a critical role in enabling families to manage care demands, with appropriate design and modifications contributing to reduced care needs and improved functioning within community settings [21]. Such adaptations often involve structural and environmental changes, including the installation of ramps, accessible pathways, and the integration of assistive products to support daily activities [22].

Assistive products, as defined by the World Health Organization, are “any product, instrument, equipment or technology adapted or specially designed to improve the functioning of a person with disability” [23]. The use of assistive products is associated with reduced inequalities, enhanced productivity, and increased participation in daily life among older adults and persons with disabilities [24]. More broadly, assistive technology, encompassing both products and related services, plays a vital role in promoting health, independence, and dignity, enabling individuals to engage more fully in educational, occupational, and social domains.

Access to home adaptations and assistive products in low-resource settings is shaped by a complex interplay of environmental, economic, and social factors. Evidence from rural South India highlights persistent barriers, including social stigma associated with disability, high costs of assistive devices, and limited availability of essential supplies, all of which restrict equitable access to rehabilitation services [25]. Similarly, the study on persons with intellectual disabilities indicates that limited awareness of assistive products, combined with insufficient funding mechanisms and a lack of proactive assessment by health professionals, further constrains appropriate utilization of these technologies [26]. Collectively, these findings suggest that both structural and knowledge-related barriers play a critical role in limiting access to home adaptations and assistive products.

Beyond access, the design of the home environment itself is a key determinant of independence and successful community reintegration. Studies emphasized that incorporating accessible housing features such as step-free entrances, walk-in showers, and ground floor living spaces can facilitate smoother transitions from hospital to home and reduce the need for costly retrofitting [27]. Furthermore, evidence from intervention studies demonstrates that home modification programs can significantly enhance community participation among individuals with mobility impairments, with reported increases in social and recreational engagement following improvements in home usability [28]. However, the sustainability of these benefits remains a concern, as gains in participation may diminish over time without ongoing support.

In the Thai context, similar challenges are evident, particularly in rural and underserved areas. Previous studies have consistently reported limited access to assistive devices, environmental modifications, and broader health care support services for persons with disabilities [29-31]. These limitations are compounded by gaps in disability registration systems and low uptake of disability-related benefits, reflecting broader systemic and policy-level constraints [32]. Although community-based home modification programs in Thailand have reported positive outcomes such as reduced activity limitations and enhanced quality of life, the underlying problems and access barriers remain insufficiently understood. This gap is particularly evident in geographically remote areas of northern Thailand, where limited accessibility continues to constrain in-depth exploration. It is crucial to identify the actual needs of persons with disabilities regarding home adaptations and assistive products to improve their quality of life, particularly in rural areas of Thailand.

The Doilor subdistrict, located in Chiang Mai Province, provides a representative example of a rural agricultural community in northern Thailand. The area comprises 26 villages with a total population of 12,227 people, the majority of whom (approximately 70%) are engaged in agriculture, including the production of longan, rice, cantaloupe, tomato, and pumpkin [33]. In 2024, there were 738 persons with disabilities in this subdistrict, accounting for approximately 6% of the total population [34]. The geographic and socioeconomic characteristics of this community may further influence access to home adaptations and assistive product services.

Previous studies in Thailand showed evidence that predominantly focused on service provision, policy implementation, and measurable outcomes. Consequently, there remains a limited understanding of how persons with disabilities, particularly in rural communities, perceive, access, and use these supports in their everyday lives. This gap is further compounded by insufficient attention to contextual factors, such as geographic isolation, seasonal transportation barriers, and local sociocultural conditions, which may significantly shape service accessibility and usability. This issue is particularly pronounced in rural northern Thailand, where access to services may differ substantially from urban areas and other regions of the country.

Addressing this gap, this study aims to explore the perspectives and lived experiences of persons with disabilities in the Doilor community regarding home adaptations and assistive products, with a specific focus on identifying barriers and unmet needs that influence their occupational engagement within home and community environments. By generating contextually grounded insights, the findings are expected to inform the development of targeted, locally relevant, and sustainable strategies to promote service provision and ultimately enhance the quality of life for persons with disabilities in rural northern Thailand.

## Methods

### Study Design

A descriptive qualitative approach [35] was conducted to investigate the experiences and perspectives of persons with disabilities toward home adaptation and assistive products. The research design, methodology, and methods were carefully aligned to ensure rigor and analytical depth [36].

### Ethical Considerations

The study obtained ethics approval from the Ethics Committee of the Faculty of Associated Medical Sciences, Chiang Mai University (reference AMSEC-65EX-030). Prior to participation, all individuals provided written informed consent, which was obtained from the participants’ legally authorized representatives prior to the study. Each participant recieved US $10 and a small gift as compensation for thier time. Interviews were audio-recorded, transcribed verbatim, and lasted approximately 1 hour. The research process was systematically documented, and transcripts were reviewed by the research team. Pseudonyms were assigned to ensure participant confidentiality.

### Participants and Recruitment

The participants were recruited on the basis of the following criteria: (1) possession of a disability identification card, (2) household registration in the Doilor subdistrict, (3) older than 18 years, (4) ability to communicate and experience home adaptations and assistive products, and (5) willingness to participate in the interview. To recruit participants, the research team disseminated advertisements over a 2-month period. These advertisements were placed in key locations within the Doilor subdistrict, including at a subdistrict health-promoting hospital, community halls, and the Doilor Community Rehabilitation Center, which is part of the Subdistrict Administrative Organization. The sample size was guided by the concept of information power as described by Malterud et al [37], whereby the adequacy of participants is determined by the relevance and data saturation in relation to the study aims and questions. Accordingly, we planned to recruit approximately 8‐12 participants who met the inclusion criteria. Individuals who expressed interest after seeing the advertisements were then contacted by the research team and assessed for eligibility based on the predefined criteria.

### Interview Procedure

The interview guide was developed based on a review of relevant literature and aligned with the study aims. An iterative process was used to refine the interview protocol, including pilot interviews conducted prior to data collection to ensure alignment between the study context, participant selection, and interview procedures. Participants for the pilot phase were purposively selected to reflect the characteristics of the target population, and the preliminary interview guide was designed to capture key concepts relevant to the study. Prompts were incorporated to facilitate in-depth responses when needed. Feedback from pilot participants and the pilot process informed further refinement of the semistructured interview guide.

In addition, the interview guide was designed with a primary focus on exploring barriers and challenges related to the use of home adaptations and assistive products, which have influenced the nature of the findings. However, participants were also provided with opportunities to share their experiences and facilitators where relevant, and these were considered during data analysis to ensure balanced representation. The first author (AK) received training in qualitative interviewing techniques prior to data collection. Face-to-face individual interviews were conducted by the first author. This included a 45- to 60-minute mock interview conducted in a private setting, which served both as a skills assessment and as an opportunity to receive feedback. All interviews were conducted face-to-face, typically in participants’ homes, to promote comfort and create a relaxed environment conducive to open sharing.

Furthermore, the research team comprises occupational therapy researchers with extensive experience in home adaptations and assistive products in Northern Thailand. Their professional backgrounds and prior engagement with persons with disabilities may have influenced data collection and interpretation. To address this, reflexive practices, including memo writing and regular team discussions, were used throughout the research process to enhance credibility and minimize potential bias. A purposive sampling strategy was used to recruit individuals with lived experience in using home adaptations and assistive products, based on predefined inclusion criteria. Participant characteristics are described in detail to enhance transparency and reproducibility. The semistructured interview questions are presented in Textbox 1.

Textbox 1. **Examples of interview questions.**

**Questions**

1. Have you ever modified your home or environment? If so, who helped you with the modifications (family, government, foundation, or private sector)? What modifications did you make? Please tell us about your experience.
2. What are the challenges you have faced in modifying your home or environment? What are the obstacles that have prevented you from making the changes you want?
3. If you had the opportunity to make further modifications to your home or environment, what would you change and why?
4. What assistive products do you use (nonslip mats, wheelchairs, hearing aids, adjustable beds, canes, walkers, wheelchairs, shower chairs, turning pillows, air mattresses, etc)? How have these products helped you? Please share your experiences with us.
5. What are the barriers or difficulties you have faced in using assistive products?
6. If you had the opportunity to choose new assistive products, what would you choose and why?

### Data Collection

Data collection occurred between December 2023 and February 2024 to generate in-depth qualitative insights. Participants who met the inclusion criteria were recruited. Semistructured interview guidelines were used, and interviews were scheduled at a time and location convenient for participants, typically conducted face-to-face by a trained researcher.

### Data Analysis

The interview data were analyzed using thematic analysis [38]. The process involved 3 researchers (HA, JG, and PM) and 2 research assistants (PT and SS) and followed a 6-step analytical procedure. First, transcripts were read multiple times to achieve familiarization and to develop a comprehensive understanding of participants’ experiences using home adaptations and assistive products. Initial notes were recorded during this stage. Second, meaningful units of text (eg, phrases or sentences) were identified and coded. Two researchers (HA and PM) independently conducted initial coding for each transcript to capture diverse interpretive perspectives. A consensus-based approach was subsequently applied, with regular meetings held to compare codes, resolve discrepancies, and refine code definitions. Third, codes were systematically organized into categories and then grouped into potential themes by identifying patterns and relationships across the dataset. For example, codes such as “I don’t have enough money” and “I have no income” were grouped under the subtheme “Insufficient financial resources,” within the main theme “Financial and personal burdens of home adaptation.” Fourth, main themes and subthemes were reviewed and refined to ensure that they accurately represented the data and aligned with the research questions. Investigator triangulation was applied throughout this stage, with multiple researchers involved in reviewing, comparing, and discussing emerging interpretations to enhance credibility. Fifth, themes were clearly defined and named to reflect their underlying meanings. Finally, the results of the analysis were reported in a written extract of the stories revealed by the analysis process.

Hence, the first and second authors (AK and HA) developed the initial version of the coding that contained the list of codes, subthemes, and main themes, which was then reviewed by the research team. Differences in interpretation were discussed and resolved through iterative team discussions, and the coding framework was refined accordingly. Participant quotations were anonymized using identification codes to ensure confidentiality.

Regarding translation, all interviews were transcribed verbatim in Thai, and coding and theme development were conducted in the original language to preserve meaning. Following the completion of the analysis, the finalized codes, themes, and illustrative excerpts were translated into English [39]. The translations were verified by 2 bilingual experts to ensure linguistic and conceptual accuracy [40]. These revisions improve the transparency, coherence, and rigor of the analytical process.

### Rigor and Trustworthiness

The evaluation of credibility, transferability, dependability, and confirmability followed the methodology outlined by Hanson et al [41]. Triangulation was used to ensure credibility. This method included interviews with 11 participants who offered diverse perspectives. The interviews were conducted following a structured protocol to maintain consistency. Transferability was assured by providing a thorough description of the sample, setting, interview questions, and findings. Additional information was made accessible to future researchers upon request to facilitate understanding and to apply the findings in different contexts.

Dependability was established through rigorous practices such as multiple researchers independently analyzing the data, peer-debriefing sessions where insights were discussed, and member checking to validate themes with participants. These steps ensured that the study’s conclusions were grounded in the data collected. Confirmability was achieved through accurate documentation: all interviews were recorded and transcribed, and researchers held regular meetings to discuss coding and thematic analysis. A final decision-making meeting involved all the researchers to ensure transparency and coherence in the findings, allowing external scrutiny and validation of the study’s interpretations. To support interpretation and analysis, a trustworthiness audit trail was established by the research team, ensuring transparency and traceability of decisions made throughout the research process [42].

## Results

### Overview

Eleven persons with disabilities agreed to participate (3 male and 8 female participants), and all of them signed consent forms before participating in the study. Pseudonyms were used to protect their identity. The demographic characteristics of the participants are presented in Table 1.

**Table 1.** Demographic characteristics of the participants in the individual interviews.

Participants	Sex	Age (years)	Cause of mobility disabled	Work status	Living situation
Tida	Female	70	Lumbar spondylolisthesis	Unemployed	Resident with family
Nawat	Male	50	Above-knee amputation	Farmer/Freelance	Resident with mother
Malee	Female	62	Congenital deformities of the hands and feet	Farmer/Freelance	Resident with siblings
Suchitra	Female	68	Below-knee amputation	Vegetable vendor	Owner and live alone
Yupin	Female	61	Ischemic stroke (left hemiplegia)	Unemployed	Resident with husband
Petchara	Female	54	Ischemic stroke (left hemiplegia)	Unemployed	Resident with family
Intira	Female	57	Ischemic stroke (left hemiparesis)	Unemployed	Resident with family
Nissa	Female	72	Lumbar spondylolisthesis	Farmer/Freelance	Resident with family
Mukda	Female	90	Femur fracture repair	Farmer/Gardener	Resident with sons
Anada	Male	76	Ischemic stroke (right hemiparesis)	Farmer/Gardener	Resident with family
Boonchu	Male	53	Hemorrhagic stroke (left hemiplegia)	Unemployed	Resident with family

Five themes arose from the analysis of the interview data: (1) financial and personal burdens of home adaptations, (2) living with safety risks in the home environment, (3) the need for assistive products aligned with individual health conditions and contexts, (4) challenges in accessing mobility support and essential services, and (5) emotional impact and sociocultural support. These themes are presented with illustrative quotes (names are pseudonyms).

### Theme 1: Financial and Personal Burdens of Home Adaptations

Persons with disabilities often face major hurdles in achieving full mobility and independence both at home and in their communities. In the rural areas of northern Thailand, persons with disabilities face unique challenges due to financial limitations. Furthermore, the participants’ situations highlight the insufficiency of government aid to cover both basic living expenses and necessary home modifications or assistive devices. In Tida’s account, she independently made necessary modifications, underscoring the challenges and costs involved in improving her daily living conditions, as she states:

*I had to handle this home modification on my own, without any help from the government. Yet, the changes have truly made my daily life much easier*.

[Tida]

Intira’s interview highlighted significant structural issues within her home that created challenges and safety risks when she used her bathroom. This underscores the difficulties in navigating various areas of the home due to these constraints, which necessitate personal effort and often incur substantial costs to resolve, as she states:

*My home has some structural problems, such as narrow and high entryways to the bathroom, which make it hard to access and increase the risk of accidents. The bathroom is important to us, so my family and I put in the effort to replace the toilet and redo the floor ourselves*.

[Intira]

Petchara’s interview illustrates the direct impact of limited financial resources on her ability to address her home modification needs, despite receiving multiple sources of government aid. This highlights a broader issue where government support is inadequate to cover both basic living expenses and the additional costs of modifying living spaces to accommodate specific needs, as the following quote reveals:

*I do not have enough money to make changes to my home. I get 800 baht a month from disability benefits, plus 300 baht from government aid. I also get 700 baht from old age benefits, so that is 1,800 baht a month in total. I need to use this money for food and bills*.

[Petchara]

Like Petchara, Yupin highlights the financial struggles of relying solely on disability and older adult allowances with little extra income. This situation presents a challenge for those with limited resources, as these allowances fall short of covering essential home improvements and daily living equipment, as she states:

*Right now, I don’t have any income from work and mostly rely on disability allowances. My husband’s income from agricultural labor isn’t enough to help us modify our home or buy the assistive equipment we need*.

[Yupin]

The financial burden of home modifications and necessary equipment is significant for individuals. Tida’s situation illustrates the limited government support, which provided a toilet without covering additional equipment costs. This highlights a common issue where incomplete government assistance forces individuals to shoulder substantial expenses, as she states:

*Even though the government gave me a toilet, I had to pay for the installation and other necessary equipment myself, which ended up costing a lot*.

[Tida]

Nissa’s account underscores the high costs of renovating essential areas such as the toilet and bathroom. Many labor costs are prohibitively expensive, illustrating the gap between the need for home modifications and available financial support, as the following statement reveals:

*The big problem is that I don’t have enough money to renovate my toilet and bathroom. Construction and hiring workers are expensive, and the labor cost is approximately 400-500 baht a day. Unfortunately, I can’t afford these expenses*.

[Nissa]

### Theme 2: Living With Safety Risks in the Home and Environment

The adaptations needed within the home, such as proper lighting, structural modifications, and assistive devices, ensure safety and comfort. The safety hazards present in the home environment include inadequate modifications and poor conditions.

In Nissa’s interview, she emphasized the need for broader home renovations to increase accessibility and improve daily living conditions. She highlights the importance of modifications such as replacing low squat toilets with higher ones and installing grab bars to address specific needs, as she recounts:

*I really want to renovate my house, especially the toilet and bathroom, to make my daily life easier. The toilet I have is now too low, so it is uncomfortable for me to use. I need a new, taller toilet that works better for me*.

[Nissa]

Similarly, Nawat’s account highlights the benefits of installing grab bars for added support and stability. This shows how grab bars can promote safer movement and greater independence at home, reducing the risk of falls and injuries. He states:

*If I got grab bars, it would be way better. It would give me extra support and stability, making it easier to stand up and move around safely, especially in the bathroom where it is easy to slip and fall*.

[Nawat]

Petchara’s account highlights the high-risk areas in her home and the surrounding environment, particularly the rough pathways with holes and bumps that promote falls. It emphasizes the challenges faced by mobility aid users and the urgent need for pathway improvements to ensure safety, as she recounts:

*The pathways around here are really rough, with lots of holes and bumps. Yesterday, I was using my walker and almost fell because of it*.

[Petchara]

Intira’s case highlights safety concerns related to poor lighting and high steps within the home. This finding illustrates the difficulties and risks associated with navigating the home environment without adequate lighting and support, leading to frequent falls and the need for home modifications to improve safety and accessibility, as she states:

*It is tough to move around my house safely without good lighting. I have fallen a few times because the steps are too high and there’s nothing to hold onto*.

[Intira]

Tida’s account emphasizes the importance of installing grab bars in key areas of the home, such as the bathroom, to increase stability and prevent falls. Grab bars provide essential support for individuals with mobility issues, helping them maintain balance and safely maneuver within their environment. This adaptation is a simple yet effective measure for enhancing safety and independence within the home, as she recounts:

*A grab bar would truly help with my stability and safety, especially in the bathroom where it’s easy to slip. Right now, I feel truly unsteady getting in and out of the shower or using the toilet because there’s nothing to hold onto. The floors can be slippery, and I often lose my balance. Installing grab bars would give me the extra support I need to move around more confidently and safely, reducing the risk of falls*.

[Tida]

### Theme 3: The Need for Assistive Products Aligned With Individual Health Conditions and Contexts

This study addresses how health issues related to disabilities significantly affect individuals’ daily activities. This highlights the physical challenges, such as pain and weakness, that necessitate aids and therapeutic interventions. The shared experiences illustrate how these conditions can limit independence and require modifications in daily routines to maintain functionality.

Health conditions associated with functional impairments directly affect physical, psychological, and social capacities, thereby increasing the need for additional support and assistive products. Mukda’s account illustrates how leg pain and difficulty walking limit her mobility, reduce functional ability, and necessitate assistance in daily activities, as she states:

*I have been dealing with some health problems, such as truly bad pain in my legs and I have a hard time walking. It has been tough because leg pain makes it hard to get around and do everyday things*.

[Mukda]

In Boonchu’s experience, this highlights the severe impact of a stroke on physical abilities and the recovery process. The use of a walker and cane, along with therapy, represents a crucial adaptation for regaining independence. This underscores the need for specific mobility aids and therapeutic support to address the challenges posed by serious health events such as strokes, as he states:

*After my stroke, I struggled with a lot of weaknesses. However, thanks to therapy and practicing with a walker and cane, I have regained more independence in my daily activities*.

[Boonchu]

The challenges and adjustments needed to transition to specific aids, such as wheelchairs, canes, and grab bars, are significant. Anada’s experience captures the difficulties individuals face when moving from a cane or walker to a wheelchair, highlighting the physical and psychological adaptations required owing to limitations with the previous aid, as described:

*I cannot use a cane because I found it too heavy and hard to manage. After hip surgery, I used a walker, but it is very hard to stand up and use it. Therefore, I have had to stop using it altogether. I'm hoping to start using a wheelchair and practice with it until I get the hang of it*.

[Anada]

Malee’s account highlights the significant benefits of an electric wheelchair for an individual’s independence and mobility. It emphasizes how a wheelchair can transform daily activities, providing a sense of freedom and comfort that other mobility aids may lack, as she recounts:

*I used to have an electric wheelchair, which was very good and allowed me to go far. I even went to buy food near the three-way intersection in the village. Currently, wheelchairs are broken and cannot be fixed. If I got a new electric wheelchair, I could help myself more, as it gives me freedom and comfort*.

[Malee]

### Theme 4: Challenges in Accessing Mobility Support and Essential Services

Access to motorized wheelchairs and other mobility aids significantly improves independence and quality of life. However, individuals face financial and practical challenges in accessing facilities and services because of mobility constraints.

Malee shared her experience of how using a wheelchair improved her sense of independence and quality of life. It highlights the contrast between life with and without a motorized wheelchair, emphasizing the newfound freedom to perform daily activities and engage in social interactions without relying on others, as she states:

*When I had a motorized wheelchair for two years, I felt a whole new level of independence compared to not having one. This wheelchair made life much easier. I could go outside, go shopping, and visit my grandkids. It was incredibly convenient*.

[Malee]

Intira reflects on the proactive efforts of individuals seeking additional mobility aids, such as canes and wheelchairs, to enhance their mobility and accessibility. This highlights the ongoing challenges faced by those with mobility issues and her determination to improve her situation through appropriate assistive devices, as she states:

*Now I am looking to get a cane and a wheelchair to help with my mobility. The cane gives me more stability and support when walking, especially on uneven ground. The wheelchair will give me a reliable way to get around, allowing me to move more freely and comfortably at home and in the community. Having these mobility aids is key for me to manage daily tasks with less difficulty and reduce the strain of walking long distances*.

[Intira]

Tida highlighted the financial challenges of modifying facilities to accommodate her mobility constraints. This underscores the gap between the basic facilities provided by the government and the additional costs individuals must bear to make these facilities fully accessible and functional for client needs, as she recounts:

*The government provided a toilet, but I had to pay for the installation and extra equipment myself, which was expensive*.

[Tida]

Suchitra indicates the daily inconvenience and discomfort experienced by individuals who do not have adequate bathroom facilities that suit their mobility needs. It emphasizes the necessity for specialized equipment, as she states:

*Currently, I sit on the toilet seat to shower, which is not very convenient. Having a shower chair that suits me would make bathing much more comfortable*.

[Suchitra]

### Theme 5: Emotional Impact and Sociocultural Support

Anxiety and fear of falling, along with dependence on others due to mobility issues, significantly impact mental health. In rural areas, individuals often rely on family and community support for essential mobility aids and daily assistance. Additionally, there is a need for support from local governments and agencies to facilitate home improvements and access to assistive devices.

Anxiety and fear are experienced by individuals with mobility issues when they are navigating uneven surfaces. Suchitra’s account emphasizes the emotional stress related to the risk of falling, which can significantly limit outdoor mobility and independence, as she states:

*I’m scared of falling when I walk, and it is truly hard if the ground outside is uneven and there’s nothing to grab onto*.

[Suchitra]

In her interview, Tida reflected on the broader mental health impacts of living with mobility constraints, especially the constant fear of falling and the need for assistance. These factors contribute to increased stress, anxiety, and a sense of helplessness, as she recalls:

*I’m scared of falling, and not being able to move around freely without help has truly affected my mental health*.

[Tida]

Like Nissa, the account illustrates the psychological relief and improved mental well-being that she anticipates from using assistive devices such as wheelchairs. This highlights the importance of mobility in reducing dependence on others and enhancing personal freedom and quality of life.

*Having a wheelchair would truly boost my mobility. I could get around on my own and go to different places without having to rely on others to carry me. It would give me more freedom and make everyday activities a lot easier*.

[Nissa]

Boonchu’s reflection highlights the influence of Thai cultural beliefs in karma (rebirth and cycles), suggesting an acceptance of disability as a consequence of actions in a past life. Additionally, Buddhist values of compassion, combined with a collectivist social structure, foster mutual support within the community, particularly for persons with disabilities, as he states.

*I truly believe in Karma. Sometimes I feel like I’m being punished for a previous life, especially since my health issues and trouble walking make it so hard to get a job. Luckily, my nephew is here to help me out. I have great neighbors, too. They help me get food from the market and never charge me a thing, simply because we treat each other like family as taught in Buddhism*.

[Boonchu]

Within the context of community support, Suchitra’s account highlights the role of the Subdistrict Administration Organization in providing essential aids, such as canes and walkers, to support individuals in their daily activities. This assistance enables the participant to engage in productive tasks, such as selling vegetables on the market, as she states:

*The staff at the local government office gave me a tripod cane and a walker to help me get to the fresh market to sell vegetables*.

[Suchitra]

Petchara’s account highlights the need for organizational support, such as local government organizations, to help people with disabilities improve their homes. This underscores the reliance on these organizations to enhance living conditions and accommodate the specific needs of those with disabilities, as she states:

*I’m looking for help from organizations such as the SAO (Subdistrict Administrative Organization) and PAO (Provincial Administrative Organization) to assist people with disabilities like me in improving our homes and living conditions*.

[Petchara]

As reflected in the aforementioned quotes, persons with disabilities often rely on government assistance, indicating that existing resources and support are insufficient to meet their needs, particularly in rural areas.

## Discussion

### Principal Findings

This study provides context-specific insights into the challenges faced by persons with disabilities in the rural Doilor community of Chiang Mai, Thailand, in relation to home adaptations and assistive products. While previous research has identified common barriers, our findings extend this knowledge by illustrating how geographic isolation, seasonal accessibility, and limited local infrastructure interact to shape everyday experiences of accessing and using these supports in a rural Northern Thai context. Consistent with prior studies, participants expressed a strong need for accessible home adaptations but faced significant financial constraints, despite partial support from local government programs. Viripiromgool et al [43] highlighted that financial barriers disproportionately affect marginalized populations in rural Thailand, while Szanton et al [44] noted limitations in formal funding systems for home modifications. Building on these findings, our study demonstrates how persons with disabilities actively navigate these constraints through a combination of limited governmental support, out-of-pocket payments, and reliance on informal community and family networks. This dynamic interplay between formal and informal support systems represents an important contribution to this study.

Semeah et al [45] reported comparable costs between rural and urban settings, with rural users incurring slightly lower expenses for bathroom modifications. Our study revealed that the cost of home modifications even in rural areas has increased due to the rising prices of construction materials and high labor costs. Furthermore, it indicates that financial pressures are more pronounced in geographically remote areas. According to Goddard et al [46], home adaptations were perceived to enhance safety, reduce physical effort, and promote independence and psychological well-being. However, our findings emphasize that these benefits remain unevenly accessible due to contextual and structural constraints. Despite these efforts, significant challenges remain, as participants and their family members continue to express concerns about how to ensure safety and effectively improve the functioning and well-being of persons with disabilities.

Addressing mobility challenges and the need for assistive products remains a critical issue. Giesbrecht et al [47] reported higher unmet needs among wheelchair users, including greater reliance on assistance and increased financial burden, while Vanderschuren et al [48] emphasized the role of structural and environmental barriers in limiting accessibility. Extending these perspectives, our study found that the assistive products should be matched to individual health conditions, functional abilities, and environmental contexts. This highlights the need for context-sensitive, person-centered approaches to assessment and provision, particularly in resource-limited rural settings. While Burns et al [49] and Sukkay and Upala [50] highlighted structural inadequacies in housing, these studies demonstrated how these limitations are compounded by local socioeconomic constraints and limited support systems, resulting in persistent safety risks such as falls despite awareness of the need for safer environments. Our findings extend previous studies by offering a context-specific understanding of how persons with disabilities in rural northern Thailand experience not only safety risks within the home but also challenges related to their surrounding environmental conditions and landscape.

In relation to mobility and service support, although previous studies have identified gaps in policy, service provision, and training [51-53], our study contributes new insight into how these systemic gaps are experienced in practice in rural settings. Specifically, we highlight the lack of repair and maintenance services, as illustrated by participants’ difficulties in maintaining essential mobility devices. Consistent with Madanhire et al [54], who highlighted the challenges faced by wheelchair users in rural areas, where rough, uneven terrain and poor road systems make it difficult for conventional wheelchairs to function effectively, our study reflected not only infrastructural limitations but also the mismatch between assistive products and the rural environment.

In addition, home adaptations are known to improve safety and reduce fall risk [55]. The study showed that it was significantly reduced 3 months after housing adaptations in the intervention group, but this effect did not persist for 6 months. Home adaptations alone were not sufficient to significantly reduce falls or near-falls. The study suggests that additional interventions are necessary to address clients’ activity limitations and improve fall-related outcomes. Our findings reveal that fear of falling and dependence on family support persist even after modifications are implemented. Hence, this study suggests that environmental adaptations alone may be insufficient, highlighting the need for comprehensive, person-centered interventions that integrate both physical provision and psychosocial support.

Importantly, this study provides novel insights into the sociocultural context. In Thailand, persons with disabilities frequently encounter social stigma and discrimination, which limit their employment opportunities. Aroonsrimorakot et al [56] reported that negative societal perceptions of persons with disabilities, often viewing them as burdensome or incapable, significantly contribute to these challenges, reinforcing barriers to both employment and participation. Consequently, limited access to job opportunities exacerbates feelings of insecurity and unease. These barriers are further intensified by difficulties in accessing workplaces, particularly among individuals with mobility impairments, which contribute to increased psychological stress [57]. Consistent with these findings, some participants in our study reported, “Right now, I don’t have any income from work and mostly rely on disability allowances.” This reflects restricted employment opportunities and a resulting dependence on government financial support.

Persons with disabilities may face limited access to employment opportunities, partly because societal perceptions in Thailand often view them as less capable of fulfilling work-related roles than individuals with no disabilities. Consistent with Jantasara [58], the societal belief that disability results from past misdeeds, prevalent in a predominantly Thai culture, further complicates the situation, making it difficult for persons with disabilities to find equitable opportunities in the workforce. However, our findings highlight the important role of sociocultural context. Cultural beliefs, including karma, Buddhist values, and a collectivist community, significantly shape how persons with disabilities accept their condition, develop coping strategies, and access support. These influences are less explicitly addressed in previous studies. Moreover, our findings demonstrate that neighbors and villagers in rural communities are often willing to accommodate and support persons with disabilities. This underscores the importance of understanding how persons with disabilities interpret and respond to disability within their local sociocultural context in rural Thailand.

Furthermore, the Ministry of Social Development and Human Security of Thailand offers subsidies for home modifications for older people and persons with disabilities. Eligible persons with disabilities can receive home assessments and improvements worth up to 40,000 Baht (US $1119) [59]. Provincial administrative organizations and local governments or subdistrict municipal offices are responsible for evaluating and making home adaptations, including installing assistive devices to meet the specific needs of individuals to help them engage in meaningful activities [60]. Our findings indicate that persons with disabilities rely on support from agencies and local government to improve their quality of life. This underscores a gap between policy and practice in rural areas, where limited funds and resources, fragmented service delivery, and inadequate implementation reduce effectiveness.

In 2023, the Economic and Social Commission for Asia and the Pacific [61] reported that Thailand has made progress in aligning national legislation with international frameworks on the rights of persons with disabilities, with notable improvements in accessibility, employment, and living standards. The involvement of organizations with persons with disabilities has been crucial in driving these legislative changes. However, persistent challenges remain, including limited interministerial coordination and the absence of systematic monitoring mechanisms.

### Limitations

This study was conducted within a single subdistrict, the Doilor community, representing a relatively limited group of persons with disabilities residing in a specific rural context. As a phenomenological inquiry, the study aimed to explore the lived experiences of persons with disabilities in relation to the use of home adaptations and assistive products within their everyday environments. While this qualitative approach enables an in-depth understanding of participants’ experiences grounded in their contextual realities, the findings may have limited transferability to other geographic or sociocultural settings. Future research should consider adopting longitudinal designs to examine the sustained and evolving impact of home adaptations and assistive products on the quality of life and occupational participation of persons with disabilities. Such approaches would allow for a more comprehensive understanding of how users’ experiences, needs, and adaptation processes develop over time within their social and environmental contexts.

### Conclusions

In summary, the experiences of persons with disabilities in rural communities in northern Thailand are multifaceted and influenced by various factors, such as financial limitations and insufficient governmental support, the home environment and safety concerns, relevant assistive devices related to disabled conditions, home adaptations, and access to assistive products. The challenges faced by persons with disabilities in these communities include barriers to accessing health care services, home modifications, and assistive products, as well as lower well-being associated with impairments. The findings suggest the need for comprehensive support systems and interventions to improve the quality of life for persons with disabilities in rural areas.

## Abbreviations

NSO: National Statistical Office
